# Novel approaches to whole sporozoite vaccination against malaria^✩^

**DOI:** 10.1016/j.vaccine.2015.09.095

**Published:** 2015-10-23

**Authors:** Else M. Bijker, Steffen Borrmann, Stefan H. Kappe, Benjamin Mordmüller, Brandon K. Sack, Shahid M. Khan

**Affiliations:** a Radboud University Medical Center, Department of Medical Microbiology, PO Box 9101, 6500 HB Nijmegen, The Netherlands; b Institute for Tropical Medicine, University of Tübingen, Tübingen, Germany; c German Centre for Infection Research, University of Tübingen, Tübingen, Germany; d Kenya Medical Research Institute-Wellcome Trust Research Programme, Kilifi, Kenya; e Seattle Biomedical Research Institute, Seattle, WA, USA; f Department of Global Health, University of Washington, Seattle, WA, USA; g Centre de Recherches Médicales de Lambaréné, Alberts Schweitzer Hospital, BP 118 Lambaréné, Gabon; h Leiden University Medical Center, Department of Parasitology, PO Box 9600, 2300 RC Leiden, The Netherlands

**Keywords:** Malaria, *Plasmodium*, Vaccine, Immunization, Sporozoite, Chemoprophylaxis, Chloroquine, Genetic attenuation, Liver stage, Pre-erythrocytic stages

## Abstract

The parasitic disease malaria threatens more than 3 billion people worldwide, resulting in more than 200 million clinical cases and almost 600,000 deaths annually. Vaccines remain crucial for prevention and ultimately eradication of infectious diseases and, for malaria, whole sporozoite based immunization has been shown to be the most effective in experimental settings. In addition to immunization with radiation-attenuated sporozoites, chemoprophylaxis and sporozoites (CPS) is a highly efficient strategy to induce sterile protection in humans. Genetically attenuated parasites (GAP) have demonstrated significant protection in rodent studies, and are now being advanced into clinical testing. This review describes the existing pre-clinical and clinical data on CPS and GAP, discusses recent developments and examines how to transform these immunization approaches into vaccine candidates for clinical development.

## 1 Background

Malaria is a life-threatening, multi-organ disease caused by blood stage infections with *Plasmodium* parasites, particularly *Plasmodium falciparum*. Disease-limiting immunity that develops after multiple malaria episodes does not prevent parasitemia or transmission [1]. WHO estimates that despite the massive roll-out of conventional control measures over the last decade, malaria causes more than 200 million clinical cases and claims almost 600,000 lives per year, mostly in African children [2]. Further progress will necessitate novel interventions that can reliably interrupt the transmission cycle and achieve elimination of malaria. A potent and long-lasting vaccine is the most promising strategy to achieve this ambitious goal; however, it has proved difficult to develop [3].

Malaria parasites are unicellular eukaryotes with a complex life cycle characterized by developmentally distinct phases of rapid and massive asexual replication in the vertebrate host and the *Anopheles* vector, linked by short but pronounced population bottlenecks during host transitions. One such bottleneck occurs during transmission, when female *Anopheles* mosquitoes inoculate tens to hundreds of motile sporozoites into the human host. Following active penetration into the blood circulation, sporozoites are passively carried to the liver where they rapidly invade hepatocytes. Within a week, a single, clinically silent round of replication from a single *P. falciparum* sporozoite will give rise to 1 × 10^3^ to 5 × 10^4^ invasive forms of the parasite, termed merozoites, which initiate the pathogenic intra-erythrocytic replication cycle and can result in up to 10^13^ parasites at the height of an infection. The small number of naturally transmitted sporozoites in an injected inoculum may explain the apparent lack of acquired pre-erythrocytic immunity even in individuals with substantial anti-blood stage immunity (as evidenced by recurrent asymptomatic and low-density blood stage re-infections) [4,5].

Experimental malaria vaccine approaches using live sporozoites and different strategies to arrest the infection *before*, *during* or *shortly after* liver stage development induce immunity targeting this clinically silent phase of the *Plasmodium* life cycle [6]. The concept is simple: deliver attenuated parasites to induce anti-sporozoite and anti-liver stage immune responses capable of reliably preventing progression of a subsequent infection beyond the liver stage and thus completely averting a pathogenic blood stage infection. Instead of trying to mimic ‘natural’ immunity against malaria parasites as induced in residents of endemic areas, whole sporozoite vaccination aims for ‘unnatural’ immunity by induction of effective, sterilizing immune responses [5,7,8]. While antibodies are important to target the extracellular sporozoites, cellular responses are likely to be crucial for elimination of intracellular liver stages. During differentiation and replication of liver stages, antigen expression changes significantly [9]. In brief, upon invasion of hepatocytes sporozoites transform inside a parasite induced cellular compartment (parasitophorous vacuole). Parasites then start a process of massive replication, termed schizogony, at the end of which, thousands of daughter cells are formed. These cells are differentiated into invasive blood stage parasites (merozoites) that are released in ‘batches’ into the blood stream within membranous remnants of their host cells (merosomes). The transformation from sporozoites to merozoites is driven by the expression of a discrete set of genes, including potential antigens. Whole sporozoite immunization therefore exposes the immune system to a wide array of parasite antigens as opposed to a single or limited number of antigens used in subunit vaccine approaches. This is an advantage because it potentially induces more potent, heterogeneous and broader immune responses that can lead to stage- and strain-transcendent immunity [10–13].

There are four approaches to whole organism immunization: radiation-attenuated sporozoites (RAS), chemoprophylaxis and sporozoites (CPS), genetically attenuated parasites (GAP) and chemically attenuated parasites. In this review we will discuss novel insights and ideas principally on CPS and GAP, whereas RAS is discussed in more detail elsewhere in this special issue.

## 2 Chemoprophylaxis and sporozoites (CPS)

Administration of sporozoites under chemoprophylaxis as an immunization method was first explored in rodent malaria models in the late 1970s–early 1980s using infection under chloroquine (CQ) prophylaxis [14–16]. The rationale is that administration of antimalarials with sporozoites allows parasite infection of the liver but prevents the erythrocytic phase where clinical disease occurs. In the case of CPS with CQ, which selectively kills asexual blood stage parasites but not sporozoites and liver stage parasites, the parasite completes liver stage development and releases infectious merozoites, which initiate the first wave of blood stages before being eliminated by the drug—exposing the immune system to multiple parasite life stages, including those parasites that either failed to enter or complete liver stage development. Though successful in animal models, the efficacy of CPS in humans was not investigated in clinical trials until 2009 when 10 volunteers were immunized 3 times by the bite of 12–15 *P. falciparum*-infected mosquitoes while receiving CQ prophylaxis [17]. This regimen resulted in sterile protection in all 10 volunteers against a controlled human malaria infection (CHMI) by mosquito bites, eight weeks after the final immunization [17]. Further, 4/6 of these ‘protected’ volunteers re-challenged at 28 months post-immunization remained fully protected—indicating a robust and prolonged immune response [18]. These results have recently been confirmed in a follow-up dose de-escalation study, showing that complete protection is dose-dependent (number of bites) and have been replicated using mefloquine (MQ), a drug with similar structure and activity to CQ, with equivalent results [19,20]. Although subjects are exposed transiently to low levels of blood stage parasitemia during CPS-CQ immunization, protection in humans appears to be heavily based on pre-erythrocytic immunity [6]. While data from animal models suggest CPS is capable of eliciting multi-stage immunity, the inability to monitor a continued parasitemia in human volunteers makes it difficult to examine the full extent of immunity against blood stage parasites [13,21–24].

Despite being logistically challenging to deliver, the dose of parasites required to generate immunity similar to RAS is considerably (orders of magnitudes) lower as just 3 × 10^−15^ mosquito bites are required for >80% protection [20] compared to the more than 1000 bites required of RAS-infected mosquitoes [25]. These encouraging and consistent results have established CPS as the current benchmark for malaria vaccine development, and have initiated a large effort to delineate immune mechanisms of protection, to generate a first-generation candidate whole-sporozoite vaccine and to optimize the CPS immunization regimen.

### 2.1 Drugs available for CPS

Using drugs targeting blood stages for CPS immunization has the advantage that full liver stage development can be achieved (as discussed in more detail later). There are many antimalarial drugs available that are candidates for CPS. They vary in efficacy, safety and mechanisms of action but only a handful have been evaluated in animal or clinical studies for use in CPS.

Drugs that suppress blood stage growth can block infections during the first round of replication inside red blood cells. The exact mechanism of one such drug, CQ, remains under debate but most likely acts by disrupting detoxification of heme, a harmful by-product of hemoglobin digestion during the trophozoite phase of blood stage infection [26]. Mefloquine (MQ) is similar to chloroquine in that it is a quinine derivative and also kills trophozoites. In order to block blood stage infection altogether, registered drugs or drug combinations known to act on replicating *Plasmodium* liver stages such as primaquine, sulfadoxine–pyrimethamine, atovaquone–proguanil, doxycycline, azithromycin and clindamycin (with the exception of primaquine, all are also potent inhibitors of asexual blood stages) could be used [27]. Primaquine, pyrimethamine and atovaquone lead to early developmental arrest, whereas the antibiotics azithromycin, clindamycin and presumably, doxycycline allow for an unconstrained production of merozoites that subsequently fail to complete erythrocytic development, due to loss of apicoplast function [28].

Thus the quiver of antimalarials available for potential use in CPS is well stocked. Although only CQ and MQ have been demonstrated effective in humans, alternative protocols have been demonstrated feasible in murine models using primaquine, pyrimethamine, artesunate, azithromycin, clindamycin and piperaquine [24,29–32]. MQ and CQ were shown to be equally effective in humans [19], and rodent studies indicate that while CPS with CQ is more potent than primaquine, antibiotics such as azithromycin induce superior immunity [31]. These findings support the hypothesis that full liver stage development, but not necessarily exposure to blood stages, is advantageous [33]. These studies not only provide greater options for clinical development, but may prove to be essential in understanding the immune mechanisms involved in CPS. Overall, the use of registered drugs with proven chemoprophylactic efficacy for CPS provides a unique platform for rapidly testing experimental whole-parasite vaccine approaches. However, in order to advance CPS from an approach to study anti-malarial immunity into a clinical intervention, further work will be required (Table 1).

## 3 Genetically attenuated parasites (GAPs)

Parasites can be attenuated by deletion of genes that are necessary at different phases of liver stage developmental progression. This attenuation strategy was made possible by the development of gene deletion methodologies in *Plasmodium* [34,35], delineation of the full *Plasmodium* genome sequence of multiple species and through transcriptional profiling. The latter identified genes that are differentially up-regulated in sporozoites and liver stages, and therefore are most likely to be essential for parasite development through the liver [9,36,37] without having a role in blood stage replication.

Genetically attenuated parasites (GAP) have demonstrated substantial to complete attenuation and sustained protective immunity in rodent malaria studies [38,39]. The first clinical study with a *P. falciparum* GAP showed considerable but incomplete attenuation [40], indicating that complete attenuation of the parasite in humans remains challenging. While GAP also face the hurdles concerning production, storage, transport and administration of other candidate live malaria vaccine approaches such as RAS and CPS, they potentially offer several benefits.

GAP are parasites with well-characterized genetic changes, while in RAS the effect of radiation acts upon the parasites’ DNA inducing non-specific damage that completely prevents parasite replication. Knowing the exact genotype allows precise characterization of the product, and provides manufacturing and regulatory advantages. Moreover, the timing of parasite arrest can be controlled in GAP, whereas RAS do not develop beyond the stage where DNA-replication is required. Consequently, GAPs that develop further into liver stage development may generate more potent immune responses. Indeed studies in rodent models with attenuated parasites have shown that immunization with greater numbers of parasites or with parasites that progress further into infection can provide superior immunity than low dose and/or early arresting parasites [11,33,41]. It has been proposed that late liver-arresting parasites have the ability to generate a greater quantity and breadth of antigen exposure that result in stronger and more robust immune responses capable of protecting against both sporozoite and blood stage challenges in murine models [11]. Equivalent immune responses are also achieved by CPS, but GAP have the advantage that they do not require the co-administration of drugs. Another advantage occurs during the manufacturing process: while RAS are fully infectious and pathogenic prior to irradiation, GAP are safe and cannot induce blood stage infection in the production staff. Finally, GAP may offer more flexibility in terms of engineering additional changes into the parasite that may increase their potency. Although this could also be done with RAS, RAS stop early into hepatic development potentially limiting expression of the introduced genes. Another advantage of GAPs is their precision of attenuation; for RAS, infected mosquitoes must be irradiated with a dose strong enough to ensure complete attenuation that is sufficient to ensure no sporozoites progress through to blood stage development. Too strong a dose of irradiation will kill sporozoites or render them non-invasive, this in turn would greatly reduce their ability induce sterile immunity, as they are ineffective at generating robust T cell-mediated immunity, which is believed to necessary for sterile immunity [31,42]. However, protocols for irradiation to produce RAS are well established and in numerous clinical trials no breakthrough blood infections have been reported.

### 3.1 Selecting gene candidates for deletion

Two features are critical to the development of a candidate malaria vaccine consisting of attenuated parasites: first and most importantly it must be safe (*i.e.* complete attenuation in the liver) and next it needs to be potent (*i.e.* generation of strong protective immunity). Therefore the foremost challenge is the selection of genes for deletion so that they create GAPs that are completely blocked in development in the liver.

To be considered a gene candidate for deletion, the gene must fulfill several criteria: The encoded protein [1] cannot be essential for blood stage replication (otherwise it cannot be deleted by current transgenic methods) [2]; it cannot have an essential function in sexual and/or mosquito stage development (otherwise sporozoites will not be produced); and [3] it must be essential for liver stage development (otherwise the GAP may progress to blood stage infection), preferably during late stages when additional target antigens are expressed and presented at high concentration.

*Plasmodium* genes that govern sporozoite and/or liver stage specific functions were first identified by characterizing mRNA transcripts that are differentially expressed in salivary gland sporozoites [36,37,43]. Deletion of some of these gene candidates (*i.e.* UIS3 and UIS4) from the genomes of rodent malaria parasites resulted in the creation of the first GAPs [44,45]. Moreover, immunization with sporozoites lacking expression of these proteins generated sterile protective immunity against infectious sporozoite challenge in mice. Independently, rodent studies investigating proteins of the *Plasmodium* 6-Cys protein family revealed that one member of this family, P52, was vital for parasite development in the liver and immunization of mice with sporozoites lacking P52 could also induce protective immunity [46]. Mutants lacking both the 6-Cys protein P52 either by itself or in combination with a deletion of the gene encoding the closely related protein, P36, were among the first GAPs to be created in *P. falciparum* [47–49]. The first phase I safety clinical trial with the *p52*^−^/*p36*^−^/*P. falciparum* GAP was conducted in 2008 where volunteers were exposed to 5 and then 200 bites from mosquitoes infected with this “double knockout” parasite. Attenuation appeared complete after exposure to 5 bites but following 200 bites, 1/6 volunteers became blood stage positive—indicating substantial but incomplete attenuation [40]. Recently, a newly identified member of the 6-Cys protein family, B9, has also been shown to be critical to parasite development in the liver and mutants lacking this protein can provoke strong protective immunity in rodent models [50,51].

After the initial identification of the first rodent malaria parasite GAP, a number of additional gene deletion mutants have been identified that are completely or partially attenuated and induce protective immunity in mice [39,52]. However, of all the GAP identified in rodent malaria parasites that could be advanced as a potential *P. falciparum* GAP, only mutants lacking the gene *sap1* (alternatively known as *slarp*), which encodes a protein involved in the regulation of transcripts in sporozoites, have been shown to fully attenuate liver stage development, even at very high sporozoite doses (up to 5 × 10^6^ inoculated intravenously) [53–55]. All other GAPs carry the risk of breakthrough blood infections dependent on sporozoite dose and mouse strain [39].

Consequently, GAP that are most advanced and currently ready for clinical evaluation consists of mutants where *slarp*/*sap1* has been removed in addition to a deletion of one or more of the 6-Cys genes from the *P. falciparum* genome. Specifically, the *P. falciparum* GAPs ‘*p52*^−^*/p36*^−^*/sap1*^−^’ and ‘Δ*b9*Δ*slarp*’ have both been examined in blood stage culture, in mosquitoes, in cultured human hepatocytes and in mice engrafted with human liver tissue and no evidence for full maturation in the liver was found [50,56]. These findings provide sufficient rationale for clinical testing to evaluate their safety and protective efficacy in humans.

The current *P. falciparum* GAPs are expected to arrest early in hepatic development, soon after invasion of hepatocytes by sporozoites. However, it has been demonstrated that immunization of mice with late liver stage-arresting GAP, lacking expression of a protein involved in fatty acid synthesis, FabB/F, elicits broader and more potent CD8T cell responses when compared to irradiated (early arresting) sporozoites [11]. This elicits superior long-lasting sterile protection against a malaria infection by sporozoite challenge and also generates stage-transcending protection against a direct blood stage challenge mediated by both T cells and antibodies [11,12]. These parasites are believed to present a greater diversity and load of parasite antigens to the host immune system. However, *P. falciparum* parasites lacking FabB/F expression fail to produce sporozoites, and thus are currently not useful for human immunization [57].

### 3.2 Genetic attenuation techniques

The permanent removal of genes from the *P. falciparum* genome is a complicated process that has required the targeted integration of plasmid constructs into the parasite genome by double crossover homologous recombination. In order to create *P. falciparum* GAPs for human use it is essential that any gene deletion is permanent and that reversion to the wild type genotype (and therefore phenotype) does not occur. Moreover GAPs must also be free of the drug-resistance markers that were used to select the deletion mutants, in order to meet regulatory concerns governing the use of genetically attenuated organisms as vaccines [50]. The removal of the drug-resistance markers has been achieved by adapting Cre and FLP recombinase methodologies, which allow for complete excision of the marker genes from the gene deletion mutant parasites [58,59]. These methods were developed in large part to facilitate the production of a multi-gene deletion candidate GAP for human use since removal of the selectable marker also makes it simpler to sequentially delete genes that govern independent biological processes. This is performed to ensure the GAP is completely (and multiply) attenuated and is thus safe for use in humans. The current ‘*p52*^−^*/p36*^−^*/sap1*^−^’ and ‘Δ*b9*Δ*slarp*’ *P. falciparum* GAP strains fulfill all these requirements [50,56] and are currently being advanced into clinical testing.

### 3.3 Novel strategies for the development of next generation GAP

The creation of a safe and protective GAP with multiple genes removed from the parasite genome could be considered a ‘first generation’ GAP. A ‘second generation GAP’ may include improvements to increase its immunogenicity, for example, one that arrests the parasite late into hepatic development and therefore induces stronger and broader protective immune responses as shown in rodent malaria models [11]. Another possibility would be to modify the parasite in such a way that its growth in culture is dependent upon a specific compound (*i.e.* an auxotroph), but importantly it is unable to develop *in vivo* in the absence of the compound. An example of such a strategy has been described where a mutant parasite has been generated that lacks a functional apicoplast and requires the addition of exogenous isopentenyl pyrophosphate for its survival in culture [60].

Moreover, existing GAPs can be further engineered, as genetic modification also allows for the insertion of genes into the genome of attenuated parasites that have been modified to be more potent. Increasing GAP potency must be directed towards generating protective immune responses with the lowest number of parasites/dose and the fewest number of doses to generate sterile protection. Genes encoding molecules that could enhance *P. falciparum* GAP potency could include (i) blood or transmission/mosquito stage *Plasmodium* antigens under the control of liver stage specific promoters to provide multi-stage immunity against malaria; (ii) genes that express proteins that could serve as adjuvants to enhance (CD8+) specific immune responses against liver stages; and (iii) genes encoding infection-limiting proteins (such as toxins) such that the GAP completely arrests late into liver stage development [61].

## 4 Chemically attenuated parasites

In addition to radiation and genetically attenuated parasites for vaccination, sporozoites that have been incubated with centanamycin, a DNA-binding drug, also induce complete liver-stage arrest in both *Plasmodium berghei* and *Plasmodium yoelii* rodent models [62,63]. Immunization with these sporozoites induces IFNγ producing CD8T cells and sporozoite specific antibodies; protection against both homologous and heterologous parasite challenge was shown to be similar to RAS [62,63]. The potential toxicity of residual centanamycin in humans is a concern, however it has been suggested that the free drug can be washed from the parasites before vaccine delivery and the drug that is present in treated sporozoites is covalently bound to parasite DNA and thus should not be available to modify host DNA [62]. This strategy is currently being evaluated for immunization with blood-stage parasites in humans (http://www.anzctr.org.au/ACTRN12614000228684.aspx), but no results have been reported so far.

## 5 Immune responses induced by whole sporozoite vaccination

Whole sporozoite vaccines face difficult hurdles in manufacturing, formulation and administration. However, they are far more effective than any alternative method investigated to date. Thus, there has been great interest in elucidating the immunological mechanisms underpinning sterilizing protection. This information can then potentially be used in the development and improvement of subunit vaccine strategies. Both cellular and humoral responses appear to play a role in protection induced by live sporozoite based immunization, although the relative contribution of each remains unclear.

Early rodent studies using RAS demonstrated an essential and predominant role for CD8T cells with a contributing but dispensable role for antibodies [64,65]. However, recent mechanistic investigations into protection by all three whole sporozoite immunization methods have demonstrated a diverse and robust immune response that encompasses both CD8 and CD4T cells as well as a significant contribution from antibodies (reviewed in [41]). Nonetheless, CD8T cells are recognized as the main effector cell in eliciting protection after whole sporozoite immunization. Cytotoxic T cells specific for the major sporozoite surface protein CSP have been shown to locate and eliminate infected hepatocytes in rodent malaria models [66]. Although the exact mechanism(s) by which CD8T cells carry out this killing is unknown, granzyme B, perforin and/or IFNγ appear to be required [41,67,68]. A recent analysis of peripheral blood mononuclear cells (PBMCs) from CPS-immunized volunteers in a clinical trial designed to elicit partial protection also implicated a strong role for cytotoxic T cells [20]. In this study, protection was correlated with CD107a expression (a marker of cytotoxic cell degranulation) on CD4 cells and granzyme B expression by CD8T cells [20]. No correlation with IFNγ expression was identified. However, this may be due to the fact that PBMCs may not mirror the makeup of liver-resident effector cells [69], which are essential for prolonged protection [70]. Analysis of PBMCs from volunteers immunized with a first generation GAP also demonstrated induction of anti-sporozoite T cell responses, associated with IFNγ-production [40]. In agreement with this clinical data, interrogation of the T cell responses in animals immunized by both GAP and CPS also indicates a critical role for CD8T cells and IFNγ for protection [11,66,71–73].

Antibodies have historically been considered dispensable for protection elicited by whole sporozoite immunization, based on studies principally performed in animal models [65,73–76]. However, these studies utilized sporozoite challenge by intravenous injection, which bypasses sporozoite passage through the dermis and minimizes the effect of antibodies on infection rate [77,78]. In a dose-escalation clinical trial with iv-administered RAS, protection in individuals correlated with the ability of serum to inhibit sporozoite invasion *in vitro* [79]. Immunization of volunteers by either CQ or MQ CPS induces antibodies that recognize both pre-erythrocytic and erythrocytic antigens but are skewed in specificity towards pre-erythrocytic antigens [19,80]. These antibodies are also highly functional as they are capable of inhibiting sporozoite infection of hepatocytes both *in vitro* and in humanized liver-chimeric mice against *P. falciparum* challenged *via* mosquito bite [81]. However, in the most recent partial-protection clinical trial of CQ-CPS, protection against mosquito bite challenge was in fact negatively correlated with anti-CSP antibody titers [82]. Although limited, data from the initial GAP clinical trial suggests that this immunization strategy is also able to elicit high titers of anti-parasite antibodies that are capable of robust inhibition of sporozoite invasion *in vitro* [83]. Indeed, recent work investigating the antibody responses to immunization of mice with a late-arresting GAP indicate that these antibodies are capable of sterile protection against a mosquito bite challenge even in the absence of T cells [84]. These antibodies also cross-react with erythrocytic stages and are capable of mediating stage-transcending protection against a blood stage challenge [24] (unpublished data, Stefan Kappe). In conclusion, the role of antibodies in whole sporozoite-induced protection is complex and seems to depend on the experimental system. Analysis of several ongoing trials may shed some more light on their protective role in humans.

## 6 Translating experimental whole sporozoite immunization approaches to vaccines

The translation of results derived from pre-clinical and clinical whole sporozoite immunization studies into a vaccine, faces considerable regulatory, manufacturing and technical challenges. Infected mosquito bite immunization is a powerful and versatile experimental tool for first-in-man studies but cannot be used for immunizing large numbers of individuals or whole populations. Therefore, future CPS or GAP vaccination will need to be performed by needle and syringe. Recently, GMP-compliant methods that meet all regulatory standards to generate and store sterile, cryopreserved and infectious *P. falciparum* sporozoites ready for use in humans have been established by Sanaria Inc., a US-based company [85]. Studies in humans, using purified and cryopreserved RAS delivered into the skin and subcutaneously by needle induced suboptimal immune responses and failed to protect volunteers against infectious malaria challenge [69]. In contrast, direct intravenous inoculation provided complete protective immunity after 5 rounds of immunization with 1.35 × 10^5^ RAS [79]. Currently, several trials are ongoing in the US, Europe and Africa to assess the effect of different immunization schedules and doses on protective efficacy (against homologous and heterologous CHMI as well as naturally acquired infection) as well as safety, tolerability and practicability of intravenous injection of sporozoites for CPS and immunization with RAS (please see the article on GMP-compliant whole sporozoite vaccines in this special issue for more details [Richie et al.])”. Optimizing the methods of sporozoite manufacturing, cryopreservation, formulation and administration is needed for whole sporozoite vaccine approaches, and requires attention by researchers and funding agencies. One possible way to improve effective immunization would be the co-administration of molecules that facilitate parasite entry into the blood or the use of adjuvants that enhance immunogenicity. For example, α-galactosylceramide, a natural killer T cell ligand, was shown to increase protective efficacy of RAS in a rodent model [86].

Another potentially transformative development is the development of an *in vitro* method of cultivating *P. falciparum* sporozoites. This would remove both the requirement for, and hand-dissection of mosquitoes for sporozoite harvest and could greatly ease the scale-up of sporozoite production—considerably reducing the cost of sporozoite-based vaccines.

## 7 Conclusions and future perspectives

The availability of GMP-grade, cryopreserved *P. falciparum* sporozoites for use in humans has ignited a series of studies that promise to move protocols quickly from phase I to phase II trials in both naïve and semi-immune volunteers and hence, closer to a potential *first-generation* product. Key milestones for future CPS and GAP studies will be (i) demonstration of heterologous protection, (ii) development of practicable regimens with injectable sporozoites and (iii) safety with regard to 100% prevention of potentially life-threatening breakthrough infections. For CPS the design of a drug regimen that supports single dose, simultaneous administration of both parasite inoculum and drug is also paramount. For GAP vaccines, the identification of *Plasmodium* genes that are critical to mid-late liver stage development and hence, the creation of late arresting *P. falciparum* GAP that are significantly more potent than early arresting GAPs would be a major improvement [9,52,87–89]. Improving the route of live-sporozoite vaccine administration is necessary for both CPS and GAP vaccines.

To meet the challenges of mass vaccination in countries where malaria is endemic it is necessary that any malaria-vaccine, in particular a live-attenuated vaccine, is safe, affordable, convenient (minimizing the number of immunizations) and it needs to generate long-lasting strain-transcending protective immunity [3,90]. While the logistical hurdles involved in delivering a live sporozoite vaccine are considerable, experimental whole sporozoite immunization regimens remain by far the most potent malaria vaccine modalities to date. Thus, in parallel with efforts to pursue licensure and delivery, careful investigation of the immune mechanisms from which they derive their potency is critical as it will direct the rational design of the next generation malaria vaccines.

## Figures and Tables

**Table 1:** Challenges and suggested lines of research for CPS and GAP.

Challenges	Lines of research
CPS	
Optimization of the prophylactic drug protocol	- Direct comparison of different drugs (*e.g.* primaquine, pyrimethamine, artesunate, azithromycin, clindamycin or piperaquine) for CPS in humans - Development of immunization with a single dose drug regimen - Re-assessment of the drug-development pipeline for novel candidates
Development of a program of mass administration using live sporozoite immunization and drug cover in malaria endemic areas	- Proof-of-concept of CPS with injectable sporozoites - Assessment of vaccine efficacy in endemic areas, and the effect of pre-existing immunity and/or (sub-clinical) blood infection - Evaluation of the effect of post-vaccination natural exposure on vaccine induced immunity
GAP	
Ensuring GAPs are completely attenuated	- Generation of multiple gene deletion mutant (each gene governing an independent and essential liver-stage function) - Generation of GAPs encoding proteins (toxins), which when expressed terminate parasite development in the liver
Optimizing GAP potency	- Generation of late arresting GAPs - Creating a GAP that re-capitulates the features of CPS (*i.e.* arrest immediately after parasite entry into the blood); possibly utilizing inducible and/or auxotrophic systems
General—whole sporozoite immunization	
Elucidation of immune mechanisms of protection	- Development of functional assays to evaluate pre-erythrocytic cellular and humoral immunity in immunized humans
Identification of key antigens and immune modulators of protection	- *In vitro* stimulation of T cells with synthetically produced overlapping peptides - Assessment of IFNγ production by T cells to recombinantly expressed or synthetic *Plasmodium* proteins (cross-) presented by autologous monocyte-derived DCs - Analysis of antibody specificities by protein microarrays - Sequencing of the B cell receptor repertoire of circulating plasmablasts and memory B cells after immunization
Generation of strain transcending immunity and improvement of durability of protection	- Immunization using multiple *Plasmodium* strains; isolation and characterization of alternative strains for CHMI and immunization - Optimal dose finding
Improvement of route of administration and	- Establish a route of administration most effective and suitable for use in large campaigns targeting young children in malaria endemic countries
Reduction of costs and improvement of practicality of whole sporozoite vaccination	- Improvement of sporozoite preservation, alteration of sporozoites; maintain viability during transport on ice or at room temperature - Establishment of axenic *in vitro* culture of sporozoites to eliminate the requirement for mosquito-based production
